# Brain Development From Newborn to Adolescence: Evaluation by Neurite Orientation Dispersion and Density Imaging

**DOI:** 10.3389/fnhum.2021.616132

**Published:** 2021-03-15

**Authors:** Xueying Zhao, Jingjing Shi, Fei Dai, Lei Wei, Boyu Zhang, Xuchen Yu, Chengyan Wang, Wenzhen Zhu, He Wang

**Affiliations:** ^1^Institute of Science and Technology for Brain-Inspired Intelligence, Fudan University, Shanghai, China; ^2^Key Laboratory of Computational Neuroscience and Brain-Inspired Intelligence, Fudan University, Ministry of Education, Shanghai, China; ^3^Department of Radiology, Tongji Hospital, Tongji Medical College, Huazhong University of Science and Technology, Wuhan, China; ^4^Human Phenome Institute, Fudan University, Shanghai, China

**Keywords:** diffusion MRI, brain development, NODDI, diffusion tensor imaging, pediatric, neurite density

## Abstract

Neurite orientation dispersion and density imaging (NODDI) is a diffusion model specifically designed for brain magnetic resonance imaging. Despite recent studies suggesting that NODDI modeling might be more sensitive to brain development than diffusion tensor imaging (DTI), these studies were limited to a relatively small age range and mainly based on the manually operated region of interest analysis. Therefore, this study applied NODDI to investigate brain development in a large sample size of 214 subjects ranging in ages from 0 to 14. The whole brain was automatically segmented into 122 regions. The maturation trajectory of each region was characterized by the time course of diffusion metrics and further quantified using nonlinear regression. The NODDI-derived metrics, neurite density index (NDI) and orientation dispersion index (ODI), increased with age. And these two metrics were superior to the DTI-derived metrics in SVM regression models of age. The NDI in white matter exhibited a more rapid growth than that in gray matter (including the cortex and deep nucleus). These diffusion indicators experienced conspicuous increases during early childhood and the growth speed slowed down in adolescence. Region-specific maturation patterns were described throughout the brain, including white matter, cortical and deep gray matter. These development patterns were evaluated and discussed on the basis of NODDI’s model assumptions. To summarize, this study verified the high sensitivity of NODDI to age over a crucial developmental period from newborn to adolescence. Moreover, the existing knowledge of brain development has been complemented, suggesting that NODDI has a potential capability in the investigation of brain development.

## Introduction

The human brain undergoes complex anatomical changes from infancy to adolescence, including axonal growth, myelination, dendritic arborization, synapse formation and neuronal pruning (Stiles and Jernigan, 2010). Comprehensive knowledge of the process of brain structure maturation is critical to understanding the cognitive and behavioral development, as well as the mechanism of the neurodevelopmental diseases, such as autism (Travers et al., 2012) and attention deficit hyperactivity disorder (Albajara Saenz et al., 2019). Valuable insights into brain development have been gained through postmortem histological explorations (Yakovlev, 1962; Gilles et al., 1983; Brody et al., 1987); nevertheless, it’s difficult to collect young healthy brain samples with a large sample size in histological studies, compared with in vivo experiments.

With the advent of Magnetic Resonance Imaging (MRI), diffusion MRI (dMRI) provides an unprecedented opportunity to measure brain anatomy in vivo. Due to the fact that dMRI can detect the microscale movement of water molecules in biological tissues, it is sensitive to pertinent microstructure and their changes. Thus, dMRI-derived metrics, such as diffusivity and fractional anisotropy (FA), have become the successful indicators of neuronal changes during brain development (Song et al., 2005). To date, the development of white matter (WM) and gray matter (GM) has been extensively studied through Diffusion Tensor Imaging (DTI). The majority of studies have demonstrated an exponential increase (e.g., *A*−*Be*^−*x*/*C*^, with A > 0, B > 0 and C > 0) in FA and an exponential decrease (e.g., *A* + *Be*^−*x*/*C*^, with A > 0, B > 0 and C > 0) in diffusivity with considerable regional variation (Mukherjee et al., 2001; Ben et al., 2005; Hermoye et al., 2006; Lebel et al., 2008; Faria et al., 2010). Both Paydar et al. (2014) and Shi et al. (2019) pointed out that except DTI, Diffusion Kurtosis Imaging (DKI) has also been applied to normal brain development by capturing the diffusion kurtosis of water molecules. In addition, DKI-derived parameters have also provided valuable insights into atypical brain development, such as preterm infants studies (Shi et al., 2016; Ouyang et al., 2019) and brain disorders like Huntington’s disease (Blockx et al., 2012).

Despite their sensitivity to brain development, both DTI and DKI models treat brain tissues as a single compartment, which is improper because the water molecules in axons, dendrites and extracellular spaces characterize different diffusion features. In order to provide more effective in vivo quantification of neural morphology, Zhang et al. (2012) proposed a compartment diffusion model termed Neurite Orientation Dispersion and Density Imaging (which can distinguish three types of microstructural environments in the brain: neurite (the collection of axons and dendrites), the extracellular space (the space around neurites), and the cerebrospinal fluid (CSF). The three measures of the NODDI model are neurite density index (NDI, also called intra-cellular volume fraction IcVF), orientation dispersion index (ODI), and isotropic volume fraction (IsoVF). Since NODDI has been proposed, emerging research has shown promising results when utilizing this compartment model in brain development studies (Chang et al., 2015; Jelescu et al., 2015; Genc et al., 2017; Mah et al., 2017; Geeraert et al., 2019). As reported by Chang et al. (2015), the correlation between NDI and age is stronger than FA from childhood to late adulthood (Chang et al., 2015). Correspondingly, Genc et al. (2017) further confirmed the sensitivity of NDI in brain development from childhood to adolescence (Genc et al., 2017). Meanwhile, Geeraert et al. (2019) included NODDI in a multiparametric analysis of white matter maturation during late childhood and adolescence, and an age-related increase was observed for NDI in a longitudinal cohort (Geeraert et al., 2019).

However, most of these studies are region of interest (ROI) based and few of them investigated the performance of NODDI metrics in gray matter development. Although Mah et al. (2017) have illustrated that NDI is strongly correlated to age in white matter and subcortical gray matter development, the results are limited to a small sample size of 27 and a relatively small age group (8–13) (Mah et al., 2017). Herein our study, we intended to apply this promising NODDI model to a reasonably large developmental dataset (*n* = 214). Compared to the previous NODDI studies, our dataset has a wider age range covered the crucial period of brain development, from newborns to young adolescence (aged 0–14). Besides, the atlas-based analysis was adopted in our research instead of the ROI-based methods. The whole brain was automatically segmented into 122 regions, including white matter, deep gray matter and cortical gray matter. Additionally, it was assumed that NODDI-derived metrics are more sensitive to age-related changes during brain development than DTI metrics, based on the observations from previous NODDI researches (Chang et al., 2015; Jelescu et al., 2015; Genc et al., 2017; Mah et al., 2017; Geeraert et al., 2019). In order to prove the feasibility of NODDI modeling in brain development, we compared the age sensitivity of NODDI and DTI metrics across the whole brain. Then, non-linear functions were utilized to evaluate the developmental trajectories of different brain regions according to the NODDI-derived NDI. And the region-specific maturation patterns were described and explained in light of different diffusion model assumptions.

## Materials and Methods

### Subjects

**FIGURE 1:**
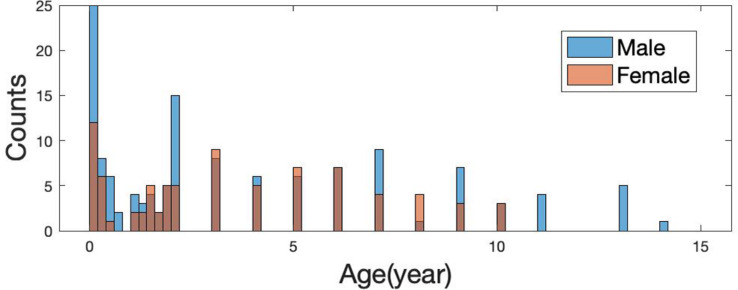
Age distribution of 214 subjects (131 males and 83 females).

The current study included 214 subjects (131 males, 83 females; ages ranging from 1 day to 14 years; age and sex distribution are shown in Figure 1) who underwent brain MR imaging for non-neurological diseases, such as oculopathy, neck hemangioma, and facial paralysis at Wuhan Children’s Hospital from November 2014 to May 2016. All subjects were born full-term. Clinical records from these participants were inspected by pediatric neurologists to ensure that no neurodevelopmental abnormalities were present. Further, this study was approved by the Wuhan City Ethics Committee of the Women and Children’s Health Care Center, and the informed consent was obtained from parents before the examination.

### MRI Acquisition

All subjects were scanned on a 3T scanner (Discovery 750, General Electric Medical System, Milwaukee, WI, United States) with an 8-channel head coil. They performed MR scanning under natural sleep or 6% chloral hydrate sedatives (0.2 ml/kg). Cotton balls and spongy pads were used for hearing protection and minimizing motion artifacts. Whole-brain dMRI was acquired along 15 directions with b = 1,000 s/mm^2^, and 15 directions with 2,000 s/mm^2^ (NEX = 20 for b = 0, NEX = 2 for b = 1,000, 2,000 s/mm^2^, TR/TE = 4,800/92.9 ms, slice thickness = 3 mm, slice space = 0, field of view = 240 mm^2^ × 240 mm^2^, matrix = 128 × 128, voxel size = 1.9 mm^3^ × 1.9 mm^3^ × 3 mm^3^, scan time = 6 min 29 s).

### Image Processing

**FIGURE 2:**
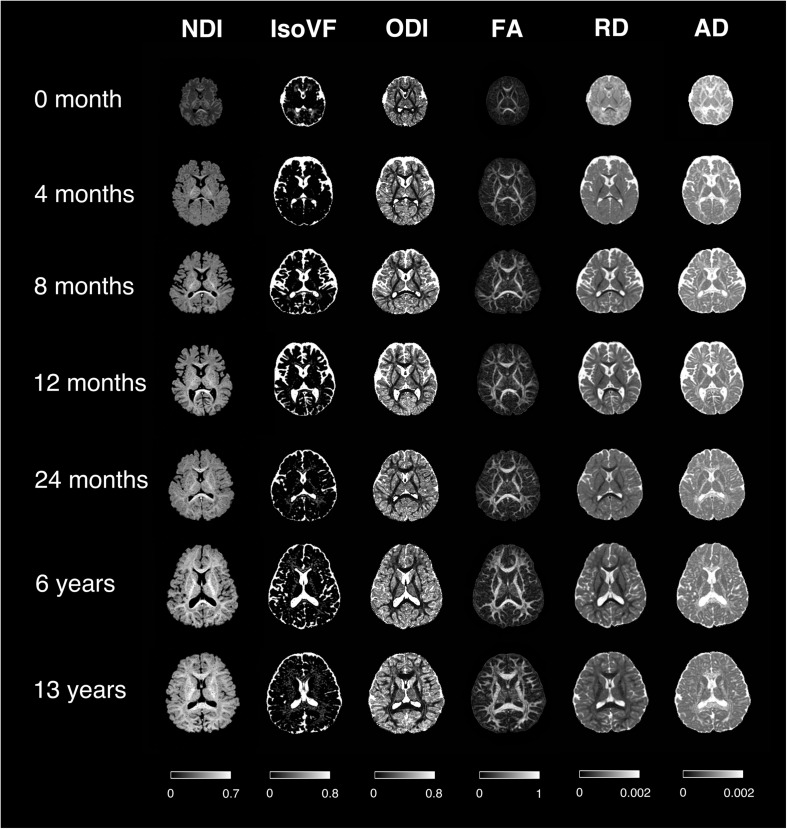
Representative mappings of NODDI and DTI-derived metrics at different ages. NDI, Neurite Density Index; IsoVF, Isotropic Volume Fraction; ODI, Orientation Dispersion Index; FA, Fractional Anisotropy; AD/RD, Axial/Radial Diffusivity. The colorbar of each column was shown at the bottom.

First of all, the raw diffusion-weighted images (DWIs) were corrected for subject motion and eddy current by the FSL *eddy* tool^1^, with b0 volume as reference for multigradient direction volumes of DWIs. The averaged head motion level can be found in Appendix Figure 1. At the same time, skull stripping was carried out based on b0 images with the FSL *bet* tool. In addition, voxel-wised diffusion metrics were obtained for each subject, including four DTI-derived metrics [FA, axial diffusivity (AD), radial diffusivity (RD), mean diffusivity (MD)] and three NODDI-derived metrics (NDI, isotropic volume fraction (IsoVF), and ODI), using the AMICO (Daducci et al., 2015) python package^2^. Especially, the NDI was calculated based on the fitted intra-cellular volume fraction (ICVF), and scaled by a factor of (1-IsoVF) to reduce the susceptibility distortion at the interface between CSF and brain tissues (see Appendix Figure 2). The scaling factor (1-IsoVF) was chosen from the original NODDI paper (Zhang et al., 2012), based on the multi-compartment model assumptions. Note that, studies have found that the default intrinsic diffusivity d_//_ = 1.7μm^2^ ms^–1^ of the NODDI model is appropriate in white matter but suboptimal in gray matter and infant brains (Kaden et al., 2016; Guerrero et al., 2019; Huynh et al., 2019). Herein our NODDI fitting, we adopted d_//_ = 1.7 μm^2^.ms^–1^ in white matter and d_//_ = 1.0 μm^2^.ms^–1^ in gray matter. And a separate set of d_//_ was used in subjects smaller than 1 month, which is d_//_ = 1.5 μm^2^.ms^–1^ in white matter and d_//_ = 1.4 μm^2^.ms^–1^ in gray matter. These d_//_ values were chosen according to the observations made by Guerrero et al. (2019). Representative maps of different diffusion metrics at different ages are illustrated in Figure 2.

### Registration

In order to improve the registration accuracy in our dataset which has a wide age range (0–14), a series of JHU Atlases^3^ were utilized for image registration, including a Neonate Atlas (Oishi et al., 2011), an 18-month Pediatric Atlas, a 24-month Pediatric Atlas and a 7-year Children Atlas (Tang et al., 2014). A total of 214 subjects were divided into 4 groups by age: (1) 50 subjects from 0 to 3 months were registered to the Neonate Atlas; (2) 41 subjects from 3 to 22 months were registered to the 18-month Pediatric Atlas; (3) 66 subjects from 22 months to 5 years were registered to the 24-month Pediatric Atlas and (4) the rest 57 subjects from 5 to 14 years old were registered to the 7-year Children Atlas. This grouped registration process is to make sure that each subject can be registered to an atlas with similar image contrast, since the image contrast of dMRI is changing along with development, especially between infants and adolescence. We adopted the dual-channel large deformation diffeomorphic metric mapping (LDDMM) algorithm for image registration by ANTs^4^, with FA and MD maps to drive the transformation (Ceritoglu et al., 2009). Each of the four atlases has its own brain segmentation, whose nomenclature followed the same adult MRI atlas (Oishi et al., 2009), based on Talairach’s atlas (Talairach and Tournoux, 1988). Note that, as described in detail in Oishi et al. (2011), the brain parcellation of neonate atlas was done manually following the adult atlas (Oishi et al., 2009) as much as possible, with careful considerations of the different brain anatomy between adults and neonates. All the four brain segmentations were projected to a standard space consisting of 122 brain regions including white matter, cortical and deep gray matter (a full name list of these 122 brain regions see Appendix Table 1). Consequently, the brain segmentation of each subject was automatically achieved after the registration.

### Evaluation of Age Sensitivity Using the SVM Regression Model

To determine whether metrics derived from NODDI and DTI in different brain regions could be adopted as a biomarker for brain development, different SVM (Support Vector Machines) regression models were tested using MATLAB. True ages were used as the training measures, and NDI, ODI, FA, and MD values from different brain regions were used as features of the SVM models. Four SVM models with different kernel functions (linear, quadratic, cubic and gaussian) were tested separately. A twofold cross-validation was adopted to evaluate the performance of the SVM models. RMSE (Root Mean Square Error) and R-Square were computed to assess the regression accuracy. Additionally, the feature weights of the linear SVM model were calculated to show the contributions of different tissue groups (WM, dGM, cGM, and others). Here, others include cerebellum and brain stems.

### Atlas-Based Quantitative Analysis

The time course of each metric, including NDI, ODI, IsoVF, FA, MD, AD, and RD, for each brain region was extracted by averaging the voxels contained in this region. In order to make a quantitative description of the developmental trajectories from different brain regions, these time courses were fitted by exponential (*Y* = *C*−*Ae*^−*age*/τ^), second-order polynomial and third-order polynomial function, separately. The goodness of fitting was assessed by R-Square and the Akaike information criterion (AIC) (Guthery et al., 2003) was used for model selection. Both metrics were compared by one-way ANOVA between the different fitting functions using Prism^5^. Afterward, the non-linear function with the best fit was chosen for the following quantification of the brain developmental pattern.

## Results

### NODDI and DTI Comparison in Age Regression

**FIGURE 3:**
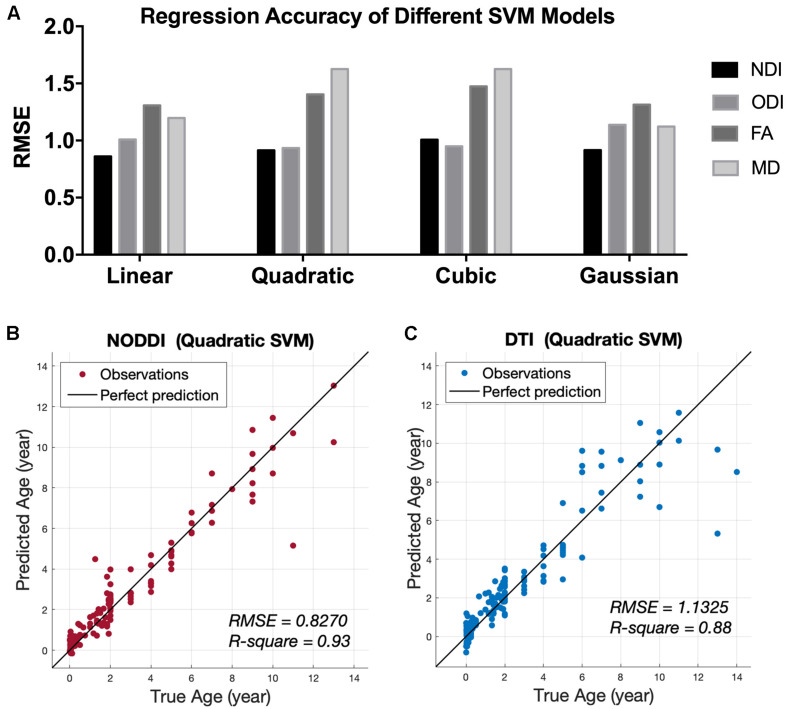
Age regression results from SVM models. **(A)** Root Mean Square Error (RMSE) of different SVM regression models between age and four diffusion metrics, NDI, ODI, FA, and MD. **(B)** Predicted age using the quadratic SVM model by the NODDI-derived metrics, NDI and ODI. **(C)** Predicted age using the quadratic SVM model by the DTI-derived metrics, FA and MD.

Representative mappings of NODDI and DTI-derived metrics at different ages were shown in Figure 2. Both NDI and FA increased with age, especially in white matter. In addition, ODI exhibited an opposite image contrast compared to FA as is expected. To quantify the age sensitivity of these diffusion metrics, SVM models were trained with NDI, ODI, FA and MD measurements, separately. The regression accuracy, assessed by RMSE, of different SVM models for each diffusion metric was displayed in Figure 3A. NODDI-derived NDI and ODI showed relatively lower RMSE than DTI-derived FA and MD in all of the four models. The age sensitivity of the two diffusion models was further illustrated in Figures 3B,C. The R-Square of age regression using NODDI-derived NDI and ODI is 0.93, which is higher than the R-Square using DTI-derived FA and MD measurements. According to the SVM evaluation above, both NODDI and DTI were sensitive to brain development, with NODDI performing slightly better. In addition, the feature weights of different brain tissue groups in the linear SVM model were summarized in Table 1 (the detailed weights of different brain regions can be found in Appendix Figure 3). As demonstrated in Table 1, NDI had more contributions in GM than WM. Besides, NDI presented more contributions in dGM regions than FA in the SVM regression on age.

**TABLE 1:** The averaged weights of features from different tissue groups using the linear SVM regression model.

		WM	cGM	dGM	Others
NODDI	NDI	0.5978	0.7325	0.7782	0.9042
	ODI	0.7314	0.7043	0.8771	1.3403
DTI	FA	0.7989	0.7854	0.4715	1.0261
	MD	0.5997	0.4393	0.7416	0.7822

*Four brain tissue groups are WM (white matter), cGM (cortical gray matter), dGM (deep gray matter) and others (including cerebellum and brain stems).*

### Quantification of NDI Development

**FIGURE 4:**
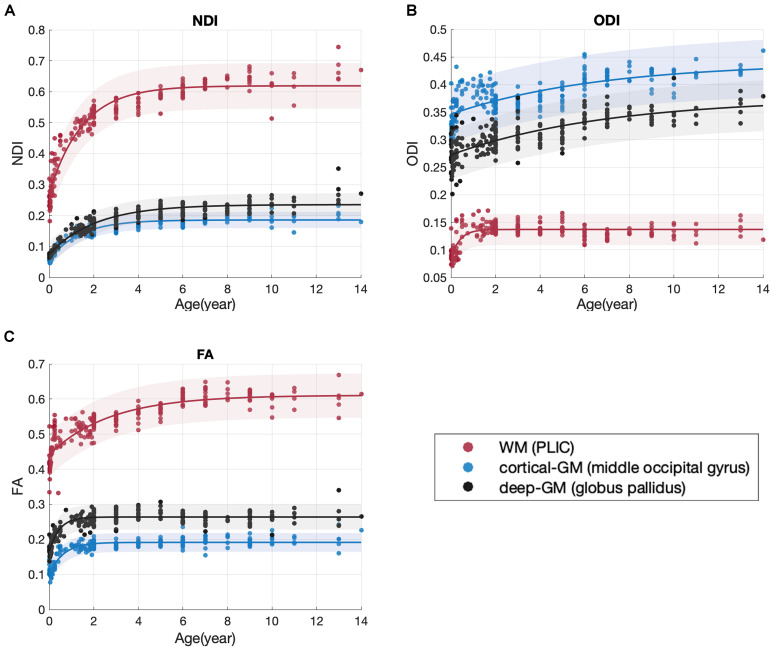
Scatter plot with the exponential fitting results of NDI **(A)**, ODI **(B),** and FA **(C)**. Three brain regions were shown as examples: the PLIC (the posterior limb of internal capsule), the middle occipital gyrus and the globus pallidus.

**FIGURE 5:**
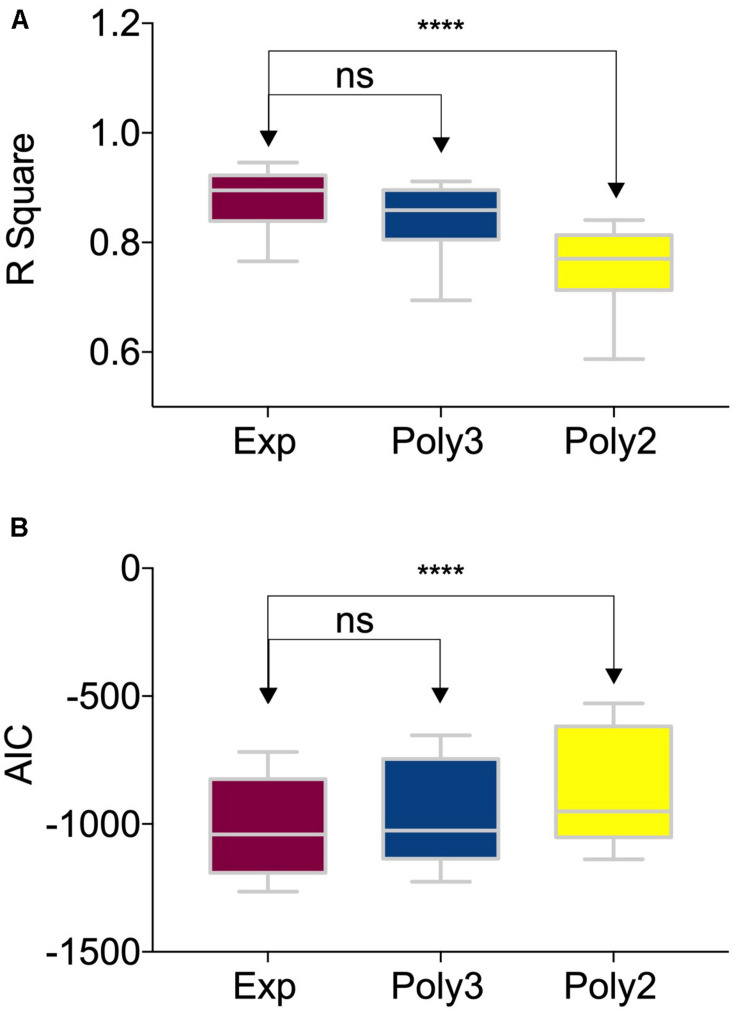
Comparison among different nonlinear fittings of NDI (Neurite Density Index) and age. The box and whiskers plot showed **(A)** the R-Square and **(B)** the AIC values of 122 brain regions using three different nonlinear fitting functions: exponential, the third-order polynomial and the second-order polynomial. The groups were compared by one-way ANOVA. The R-Square of the exponential fitting was significantly higher than that of the second-order polynomial fitting (*P* < 0.0001, denoted by ‘****’). And there was no significant difference between the exponential fitting and the third-order polynomial fitting. The one-way ANOVA test results of AIC were the same as those of the R-Square.

The developmental trajectories characterized by NDI measurement showed non-linear growth in all the 122 brain regions. Figure 4A exhibited three example brain regions, representing white matter, deep gray matter and cortex. The one-way ANOVA tests between different fitting functions were demonstrated in Figure 5. The exponential fitting showed the best fitting goodness with the highest R-Square. When model complexity was considered together with the goodness of fitting, the exponential fitting also had the best performance with the lowest AIC value. Consequently, the exponential function was selected to quantify the NDI developmental trajectories. Despite of the AIC criterion, a more comprehensive understanding of this choice could be that the exponential function has more interpretable parameters. As presented in Figure 4A, NDI increased dramatically in the first 2 years, then it slowed down and gradually plateaued. These characteristics are consistent with those of the exponential function *Y* = *C*−*Ae*^−*age*/τ^, in which the increasing speed (characterized by the first-order differential of *Y*, *dY*/*dt* = (*A*/τ)*e*^−*t*/τ^) also slows over time and the ***Y*** converges to the asymptote indicated by ***C***. Here, ***C*** and ***A*** reflect the mature value and the total growth of NDI respectively. And the time constant ***τ*** is an estimate of the time scale of NDI growth in different brain regions.

### Developmental Patterns Between Tissue Groups

**FIGURE 6:**
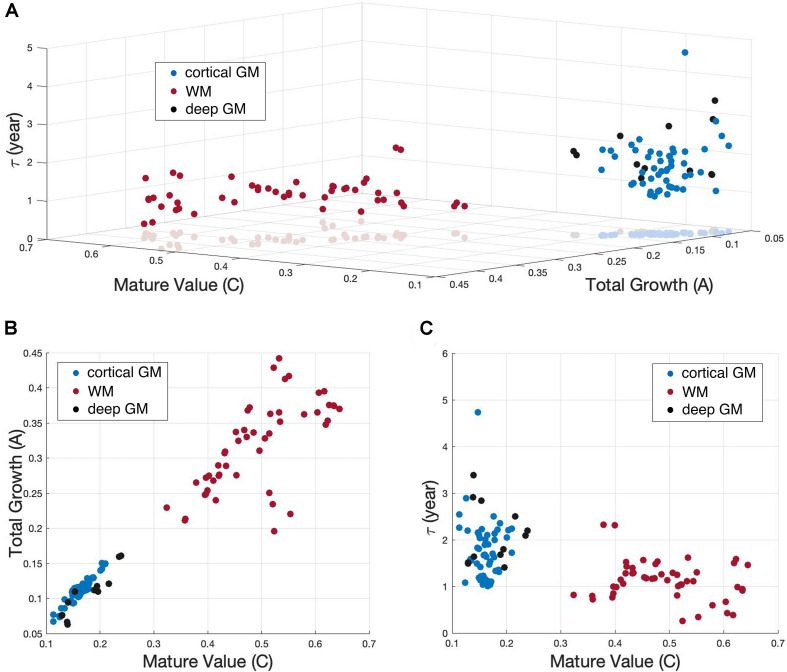
Scatterplot showing the exponential (*Y* = *C*−*Ae*^−*age*/τ^) fitting results of NDI. **(A)** 122 brain regions with the three fitting parameters (*C*, *A*, and τ) as coordinates were shown in a 3D scatterplot. White matter (WM), cortical gray matter (cortical GM) and deep gray matter (deep GM) were represented by red, blue and black dots, respectively. **(B)** 2D scatterplot exhibits the *C* versus *A* results. **(C)** 2D scatterplot exhibits the *C* versus τ results.

To investigate whether different tissue groups differ from each other in terms of the different variables (***C***, ***A***, ***τ***) of the exponential fitting, the 122 brain regions were clustered into 3 tissue groups, white matter, deep and cortical gray matter. A 3D scatterplot with ***C***, ***A*** and ***τ*** as coordinates for all the 122 brain regions was shown in Figure 6A. Brain regions in GM (including both cortex and deep gray matter) were easily distinguished from regions in WM by their ***C*** values and ***A*** values, which were more clearly shown in the 2D scatterplot in Figure 6B. Cortical GM had a lower ***C*** (P = 2.690e-10) and a lower ***A*** (P = 1.274e-7) than WM. Deep GM also had a lower ***C*** (P = 0.0034) and a lower ***A*** (P = 1.045e-4) than WM. However, the time constant ***τ*** was region-specific and showed no significant difference between groups, which can also be observed in Figure 6C.

**FIGURE 7:**
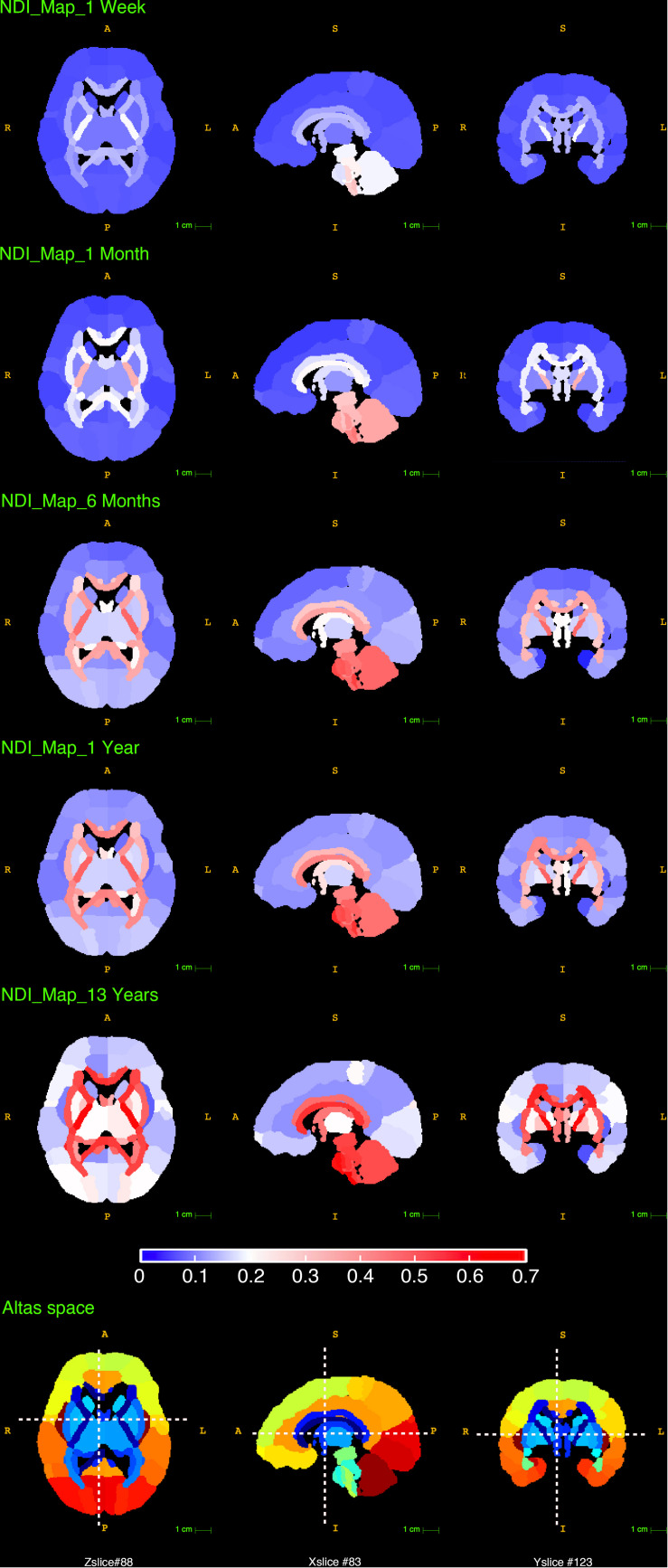
The reconstructed NDI maps at 1 week, 1 month, 6 months, 1 year and 13 years old. All the maps in this figure shared the same colorbar shown at the bottom. The last row displayed the slice numbers and the locations of each brain view shown in this figure.

Since the parameter ***A*** is an estimate of the total NDI growth from newborn to young adolescence (0∼14 in our study), the distinction in ***A*** between WM and GM found above was also examined by comparing the NDI maps of newborns and 13 years olds. In Figure 7, the top row presented the NDI maps for the newborns, which were calculated by averaging the youngest 10 subjects whose ages ranged from 1 day to 1 week old. The fifth row presented the NDI maps for the 13 years old, which were the averaged results from 5 subjects aged 13. The bright areas in the newborn NDI maps (the top row) indicated that the PLIC (the posterior limb of internal capsule), the brain stems (including medial lemniscus, medulla oblongata and pons), the cerebellar hemisphere and its related peduncles already had relatively high NDI values at birth. And there were no clear distinctions observed between WM and GM. Nevertheless, the differences between WM and GM were more pronounced in the NDI maps at 13 years of age (the fifth row), which means WM underwent more NDI growth during childhood and young adolescence. And this is in line with the higher total growth ***A*** and the higher mature value ***C*** we found in WM. The second to the fourth row in Figure 7 demonstrated these NDI changes between newborns and young adolescence at 1 month, 6 months and 1 year.

### Developmental Rate

**FIGURE 8:**
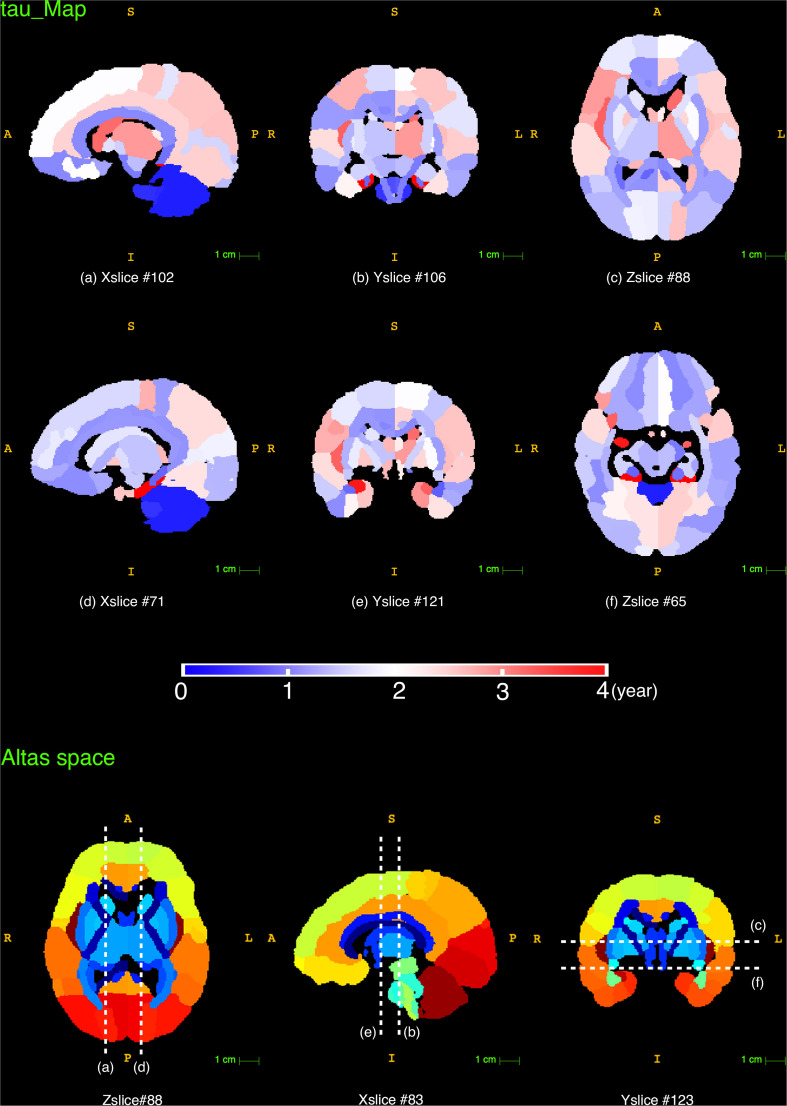
The reconstructed ***τ*** maps from the exponential fitting (*Y* = *C*−*Ae*^−*age*/τ^) of NDI measurements. The last row displays the slice numbers and the locations of each brain view shown in this figure.

**FIGURE 9:**
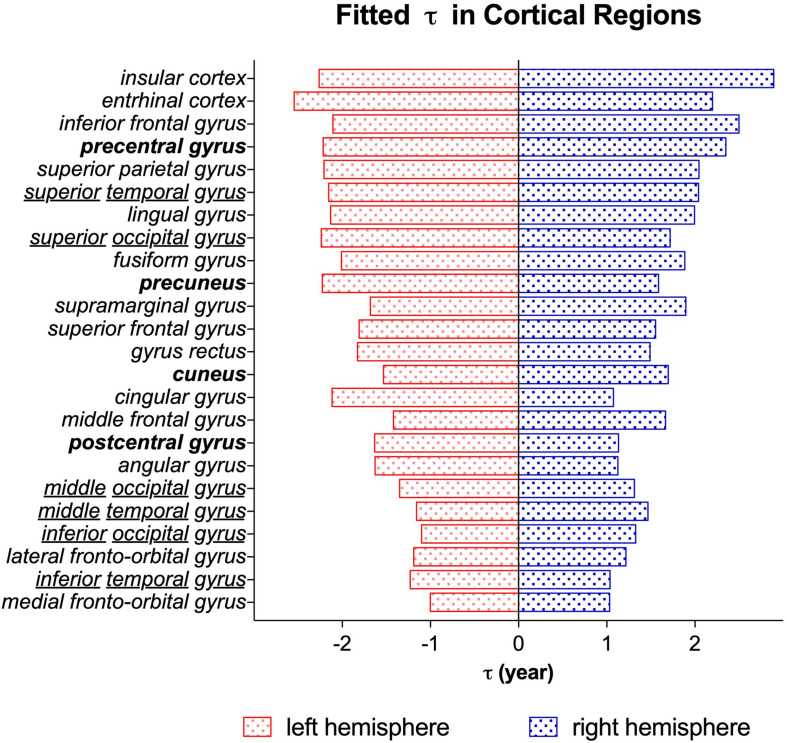
The time constant ***τ*** from the exponential fitting of NDI measurements of different cortical regions. Brain regions are listed in the descending order ***τ*** from top to bottom. The blue bar stands for the brain regions in the right hemisphere. The red bar stands for the brain regions in the left hemisphere. The brain regions highlighted in bold (postcentral and precentral gyrus, cuneus and precuneus) represented a posterior to anterior developmental trend. And the underlined regions (temporal and occipital gyrus) exhibited an inferior-middle-superior trend of development.

The time constant ***τ*** reflects the time it takes to reach the asymptote ***C***, which is a prediction of the time it takes for a brain region to reach its mature NDI. As displayed in the ***τ*** maps in Figure 8, the cerebellum had the lowest ***τ*** than other brain regions, indicating that NDI grows faster in both cerebellar hemispheres. Besides, the white matter tracts like the corpus callosum, the anterior and posterior limb of the internal capsule had relatively lower ***τ*** than cortical regions like the superior frontal gyrus, superior parietal gyrus and superior occipital gyrus. Example regions in the cortex were listed in a descending order ***τ*** from top to bottom in Figure 9. It was found that the postcentral gyrus matured faster than the precentral gyrus, the same trend was observed in the cuneus and the pre-cuneus (these regions were highlighted in bold in Figure 9). Additionally, although the frontal gyrus did not exhibit this inferior to superior gradient in NDI growth, both the temporal and the occipital gyrus showed an inferior-middle-superior development trend (the temporal and occipital gyrus were underlined in Figure 9).

## Discussion

### Whole-Brain NODDI Study From Newborn to Adolescence

This is the first study to date utilizing NODDI to investigate the maturation trajectories of the whole brain from infancy to adolescence. In previous NODDI studies, the ages of subjects were limited to 0 to 3 years (Jelescu et al., 2015) or 8 to 13 years (Mah et al., 2017). Here, we recruited subjects from 0 to 14 years old, which covered the crucial range of brain development. Hence, age-related microstructural changes of brain tissues from birth to adolescence could be investigated in one dataset. NODDI is more specific to brain tissues than the widely used DTI model. In the current study, the NODDI-derived NDI and ODI outperformed the DTI-derived metrics in SVM regression models of age, similar results have also been reported in previous studies (Chang et al., 2015; Genc et al., 2017; Mah et al., 2017). This age-related sensitivity in NODDI is beneficial from the multicompartment assumption in its model. By providing sensible neurite density and orientation dispersion estimates, NODDI disentangles the two key contributing factors (NDI and ODI) in FA (Zhang et al., 2012). Fractional anisotropy is an index for the amount of diffusion asymmetry within a voxel, which is influenced by both the orientation dispersion and the neurite density. For example, the increase of FA may be caused by the increase of neurite density or the reduction of orientation dispersion. Take the three representative brain regions in Figure 4 as examples, both NDI and ODI increased with age in these brain regions. The increase of NDI might cause an increase in FA, but the increase in ODI might reduce the FA at the same time. Therefore, the FA time course in Figure 4C actually showed the hybrid results of both NDI and ODI. This offers one possible explanation for the better age regression accuracy of NODDI metrics, suggesting that NODDI may provide more biologically specific characteristics in brain development than DTI (Chang et al., 2015). Figure 3 shows that the sensitivity of ODI to age was slightly lower than that of NDI. Both Kunz et al. (2014) and Mah et al. (2017) also indicated that the correlation between ODI and age is weaker than that between NDI and age.

### Assessment of Developmental Trajectories

In this study, the exponential function *Y* = *C*−*Ae*^−*age*/τ^ showed the best fit in the quantification of NDI developmental trajectories. Previous studies have found that the human brain experienced dramatic growth during the first two years and slowed down after 2 (Barkovich et al., 1988; Mukherjee et al., 2002; Schneider et al., 2004; Hermoye et al., 2006), in line with the features of the exponential function, whose increasing speed *dY*/*dt* = (*A*/τ)*e*^−*t*/τ^ also declines with time. Besides, two additional exponential fittings were calculated for males and females separately to test the gender effect. The Student’s *t*-test was utilized to evaluate if the distributions of the fitted parameters (***C***, ***A***, ***τ***) over 122 brain regions stay the same between males and females. The p-values of these t-tests were listed in Table 2. All the *p*-values were larger than 0.05, which means no significant difference was found between males and females based on the NDI and ODI trajectories in our study.

**TABLE 2:** Results of *t*-test between genders.

The *P*-values of gender tests			

Metrics	C	A	τ
NDI	0.146	0.147	0.517
ODI	0.305	0.306	0.326

*The exponential fitting (*Y* = *C*−*Ae*^−*age*/τ^) of NDI and ODI were applied to males and females, separately. The distribution of the obtained fitting parameters (*C*, *A*, and τ) was tested between genders, using the Student’s *t*-test.*

### Assessment of Brain Developmental Pattern

As shown in the reconstructed NDI maps (Figure 7), the cerebellar hemisphere and its related peduncles already exhibited notably high NDI at newborns, suggesting a remarkable neural development in the cerebellum before birth (Cho et al., 2011). Meanwhile, the prominently larger NDI in the brain stem and cerebellar regions at birth is consistent with the existing knowledge that the brain stem and cerebellar areas myelinate prior to the cerebral hemispheres (Barkovich et al., 1988). Additionally, the cerebellum still presented a relatively high NDI at 13 years old, agreeing with the understanding that the cerebellum contains more than half of all neurons in the human brain (Herculano-Houzel, 2010; Herculano-Houzel et al., 2015).

The group difference of variable ***C*** found between WM and GM can be explained by the assumptions of NODDI models. NDI estimates the volume fraction of both axons and dendrites (Zhang et al., 2012). Since white matter are mainly occupied by compact axon bundles, while gray matter are composite of both dendrites and cell bodies(soma), the volume fraction of neurites in gray matter is expected to be smaller than that in white matter. The other distinct variable between WM and GM groups is ***A***, which stands for the total growth of NDI value from newborn to young adolescence (0∼14). This indicates that white matter has experienced a more rapid NDI growth in childhood and early adolescence. Another view on the difference between gray and white matter development is provided by ODI. According to its original definition, ODI estimates the angular distribution of neurites. The change in ODI may reflect the axonal organization and neural pruning (Sowell et al., 2004). In our study, the ODI time courses of different brain regions reach their plateaus at distinct time points. For instance, in Figure 4B, two example GM regions, the globus pallidus and the middle occipital gyrus, underwent ODI increase throughout childhood and early adolescence. Nevertheless, the example WM region, PLIC, remained a stable ODI after 2 years old.

Based on the assumptions of the NODDI model, NDI may provide an estimate of the cerebral neurite density. Still, more studies are needed to validate the relationship between the histologic cerebral neurite density and the NODDI-derived NDI. For the assessment of NDI developmental patterns, we should be careful to draw any direct biological explanations. However, it still gives us valuable insights by comparing the NDI developmental patterns with the rich findings from histological studies. For instance, the fifth row in Figure 7 showed an averaged NDI map at young adolescence, a spatial gradient of NDI values was found in the cortex. The NDI in the precentral and postcentral gyrus were higher than those in the prefrontal, parietal and temporal areas, showing a spatial gradient that anchored in the sensorimotor regions and radiated to the surrounding areas. A similar gradient has been described in previous studies on cortical thickness (Wagstyl et al., 2015) and myelin content (Burt et al., 2018; Huntenburg et al., 2018). Meanwhile, postmortem histology studies have demonstrated a strong correlation between cerebral neurite density and the intensity of myelin stain under light microscopy (Jespersen et al., 2010). Although there is no confirmed association between the NDI gradient found in this study and the previously revealed gradients of myelin content or cortical thickness, this comparison still suggests that NODDI analysis could provide additional insight to the multi-model studies of brain development.

The time constant ***τ*** was more region-specific and no significant difference was found between white and gray matter. In cortical regions, an inferior-middle-superior trend was observed in both the temporal and the occipital gyrus, which is potentially related to the myelination process because myelination is generally thought to process from inferior to superior (Yakovlev and Lecours, 1967; Brody et al., 1987).

### Limitations

Due to most of children cannot stay motionless in the MR scanner for a long time, acquiring MRI data on pediatrics especially on infants is challenging. More difficult is that NODDI requires at least two shells of diffusion MRI data for its model fitting, which doubles the scanning time of the traditional DTI acquisition. Therefore, the number of diffusion gradient directions in our study was compromised to shorten the acquisition time for our young participants, which is lower than the recommended number of the original NODDI protocol (Zhang et al., 2012). In order to evaluate the quality of our NODDI fitting, we did a comparison between the fitting results of our image protocol (***protocol_30***: 15 directions at b = 1,000 s/mm^2^ and 15 directions at b = 2,000 s/mm^2^) and the full NODDI protocol (***protocol_90***: 30 directions at b = 1,000 s/mm^2^ and 60 directions at b = 2,000 s/mm^2^). The comparison of fitted NDI was shown in Appendix Figure 4. With the ***protocol_90*** considered as a reference, the fitted NDI of ***protocol_30*** was in good agreement with the reference in WM. In GM, the fitted NDI of ***protocol_30*** was lower than the reference with an averaged difference of −0.0167 ± 0.0106 (mean ± std). This means that the reconstructed NDI values, as well as the fitted parameter ***C*** from the exponential fitting, may be underestimated in GM in our study. Nevertheless, according to the NDI ranges in the present study, the NDI was between 0 and 0.2 in GM and 0∼0.7 in WM (see Figure 6), which means, the fitted parameter ***C*** in GM would still be distinguishable from those in WM even if there was an NDI bias in GM. The comparison of ODI was presented in Appendix Figure 5. Unlike the observations of Zhang et al. (2012) that low angular resolution will undermine the accuracy of ODI estimation while has little impact on NDI (Zhang et al., 2012), the fitted ODI of ***protocol_30*** was in line with the reference in GM. In WM, the ODI of ***protocol_30*** showed a little bias compared to the reference with an average difference of −0.0162 ± 0.0154 (mean ± std).

Additionally, the dMRI of all subjects from 0 to 14 years old were acquired using the same set of b values (0, 1,000, and 2,000 s/mm^2^). While the optimal protocol recommended for NODDI used b values = 0, 711, and 2,855 s/mm^2^ (Zhang et al., 2012), Wang et al. (2014) showed that the more standard protocol utilized here in our study yields comparable performance. Besides, considering that the neonatal brain contains more water and is less myelinated than the adult brain, the b values used in DTI studies for neonates are smaller than those for adults, which are ranged between 600 and 1,100 s/mm^2^ (Heemskerk et al., 2013). And the use of b values around 700 s/mm^2^ is believed to be optimal for infant brains (Pannek et al., 2012). Up to now, there is no consensus on the optimal b-values in the neonatal brain for NODDI, and the same set of b values (0, 1,000 and 2,000 s/mm^2^) has been used by other groups when applying NODDI to neonatal studies (Jelescu et al., 2015; Karmacharya et al., 2018). Still, given the knowledge from DTI studies, the b = 1,000 s/mm^2^ used in our study might be a little too high for infants, which may affect the SNR of the acquired dMRI data. But the influence on the fitted diffusion metrics might be small, according to the observations from the DTI study, the FA values remain stable for b values up to 3,000 s/mm^2^ in neonates (Dudink et al., 2008).

Note that, it is still necessary to process further longitudinal animal studies to verify the relationship between the NODDI-derived NDI and the histological indices, such as neurite density, neuron number and neuron density. With such verification, the physiological significance of NODDI metrics can be clearer and be used to guide future research in the human brain.

## Conclusion

This study verified NODDI’s sensitivity to brain development by quantitative comparison with the most widely used DTI model, over a large sample size (214) covering a crucial development period (0∼14 years old). NODDI outperformed DTI in SVM regression models of age. Both NDI and ODI showed a non-linear increase with age. NDI in white matter experienced higher total growth than that in gray matter (including the cortex and deep nucleus). Region-specific maturation patterns were described throughout the brain and were evaluated on the basis of model assumptions. These findings complemented the existing knowledge of brain maturation and indicated that NODDI is more specific than DTI in neural changes detection, suggesting that NODDI can be a powerful tool in further investigations in brain development, as well as aging and neurodevelopmental abnormality.

## Data Availability Statement

The original contributions presented in the study are included in the article/Supplementary Material, further inquiries can be directed to the corresponding author/s.

## Ethics Statement

The studies involving human participants were reviewed and approved by Wuhan City Ethics Committee of the Women and Children’s Health Care Center. Written informed consent to participate in this study was provided by the participants’ legal guardian/next of kin.

## Author Contributions

JS and WZ acquired the MRI data. FD did the data preprocessing. XZ did the data analysis, language editing, and wrote the manuscript. HW encouraged XZ to investigate and supervised the findings of this work. LW helped to discuss the MRI registration strategy. BZ and CW participated in the discussion of the results. XY participated in the language editing. All authors contributed to the article and approved the submitted version.

## Conflict of Interest

The authors declare that the research was conducted in the absence of any commercial or financial relationships that could be construed as a potential conflict of interest.

## Footnotes

1 https://fsl.fmrib.ox.ac.uk/fsl/fslwiki/eddy

2 https://github.com/daducci/AMICO

3 http://cmrm.med.jhmi.edu/

4 https://sourceforge.net/projects/advants/

5 https://www.graphpad.com/scientific-software/prism/

## Supplementary Material

Appendix Figure 1 Root mean square head motion (RMS_motion) of 214 subjects. The averaged RMS_motion of 40 diffusion volumes were shown with a standard error bar. The averaged RMS_motion of all subjects was 0.27mm.

Appendix Figure 2 A showcase of NDI calculation. The first row represented the calculated NDI maps. And the corresponding ICVF and IsoVF maps were shown in the second and third row. Compared to the ICVF maps, the distortion at the interface between CSF and brain tissues had been reduced in the NDI maps. All the maps in this figure shared the same colorbar shown at the bottom.

Appendix Figure 3 Scatterplot showing the feature weight of each brain region using the linear SVM regression model for **(A)** NDI, **(B)** ODI, **(C)** FA and **(D)** MD. Four brain tissue groups are WM (white matter), cGM (cortical gray matter), dGM (deep gray matter) and others (including cerebellum and brain stems).

Appendix Figure 4 NDI comparison between ***protocol_30*** and ***protocol_90***. **(A)** The fitted NDI in 38 white matter (WM) regions. **(B)** The fitted NDI in 58 gray matter (GM) regions.

Appendix Figure 5 ODI comparison between ***protocol_30*** and ***protocol_90***. **(A)** The fitted ODI in 38 white matter (WM) regions. **(B)** The fitted ODI in 58 gray matter (GM) regions.

Appendix Table 1 Exponential fitting results of NDI. * The order of brain regions remains the same as JHU Atlases (http://cmrm.med.jhmi.edu/).
