# Supplementary figures and images for: Brain Development From Newborn to Adolescence: Evaluation by Neurite Orientation Dispersion and Density Imaging
Source: Front Hum Neurosci. 2021 Mar 15;15:616132. doi: 10.3389/fnhum.2021.616132 (PMC8005551; doi:10.3389/fnhum.2021.616132)

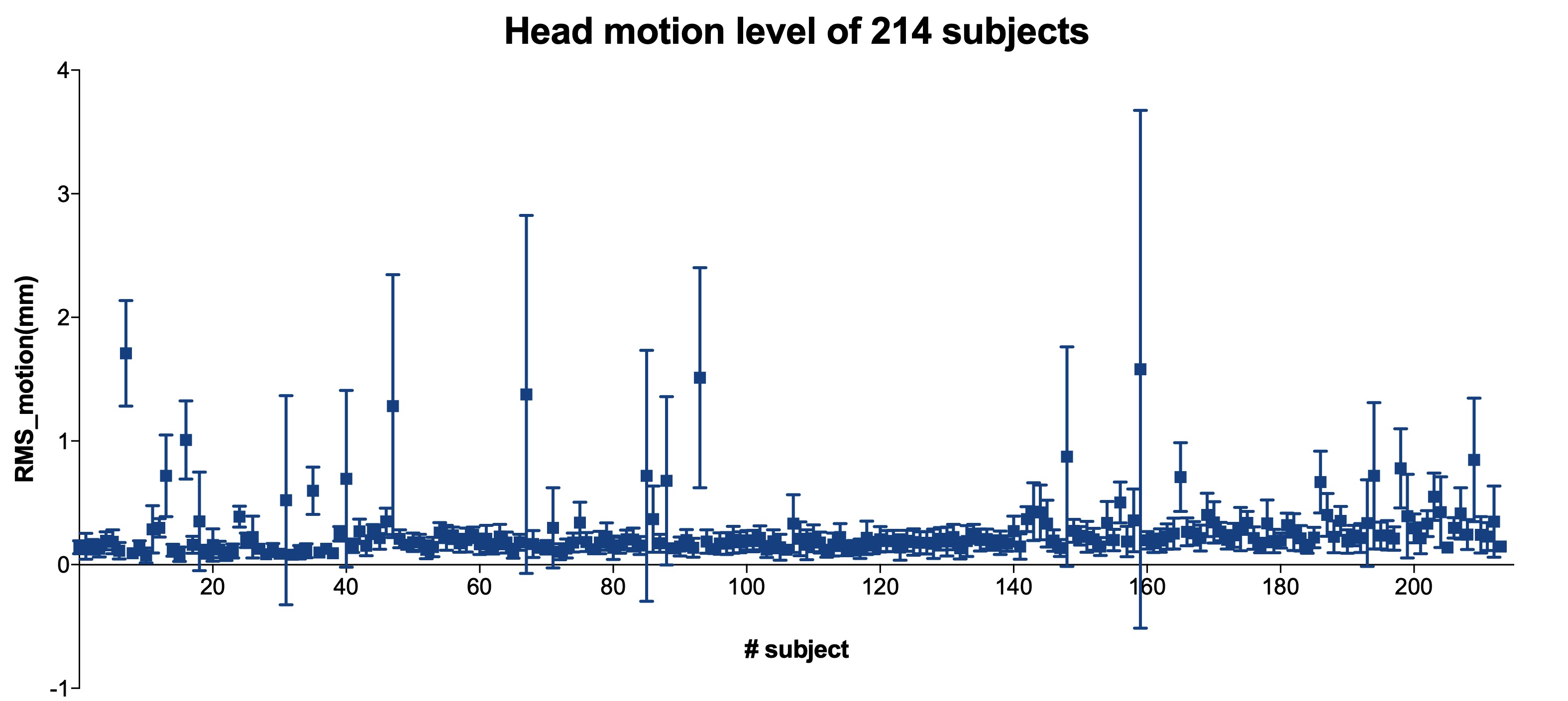

Supplement: Appendix Figure 1 — Root mean square head motion (RMS_motion) of 214 subjects. The averaged RMS_motion of 40 diffusion volumes were shown with a standard error bar. The averaged RMS_motion of all subjects was 0.27mm. [file Image_1.JPEG]

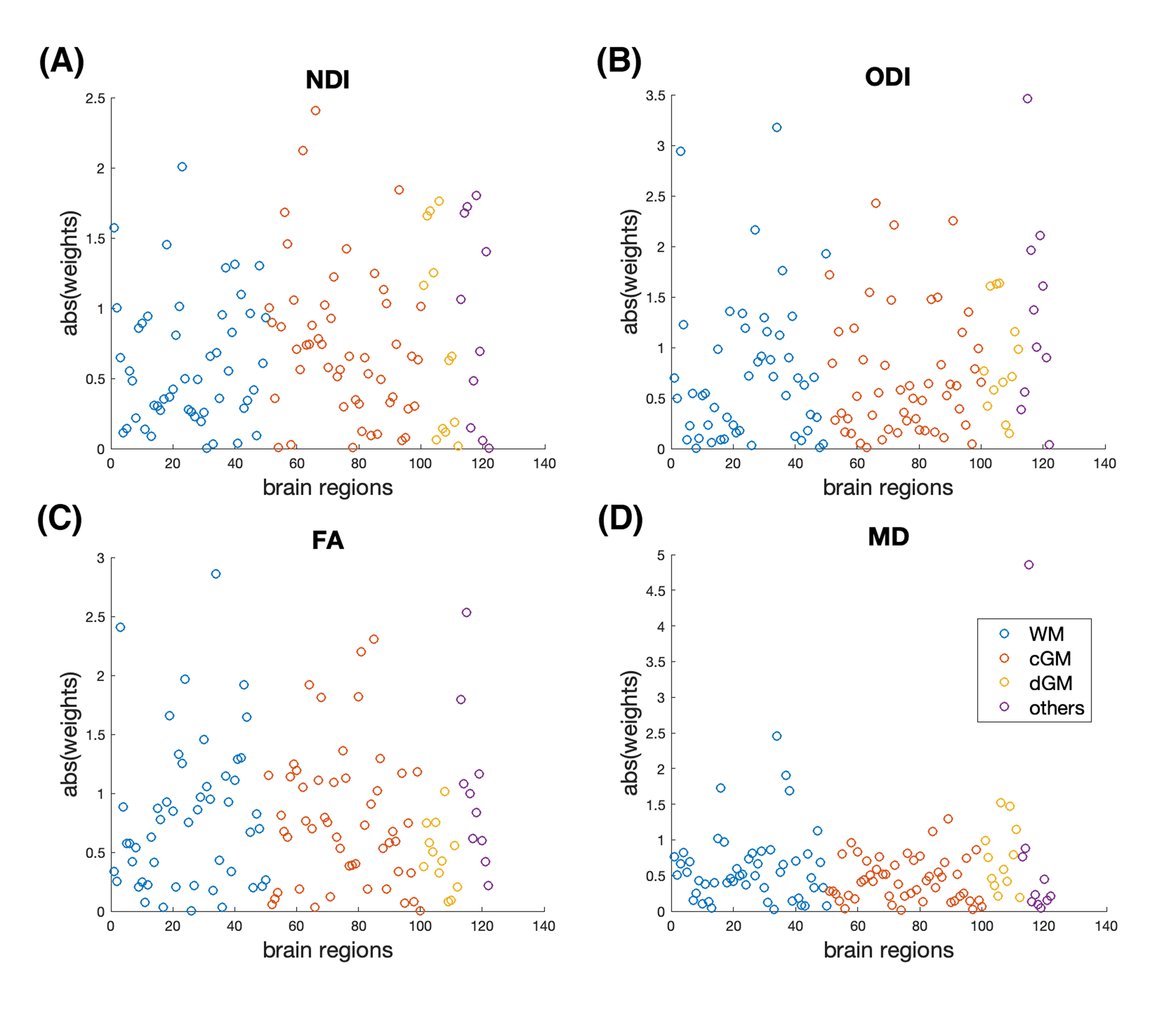

Supplement: Appendix Figure 3 — Scatterplot showing the feature weight of each brain region using the linear SVM regression model for (A) NDI, (B) ODI, (C) FA and (D) MD. Four brain tissue groups are WM (white matter), cGM (cortical gray matter), dGM (deep gray matter) and others (including cerebellum and brain stems). [file Image_3.JPEG]

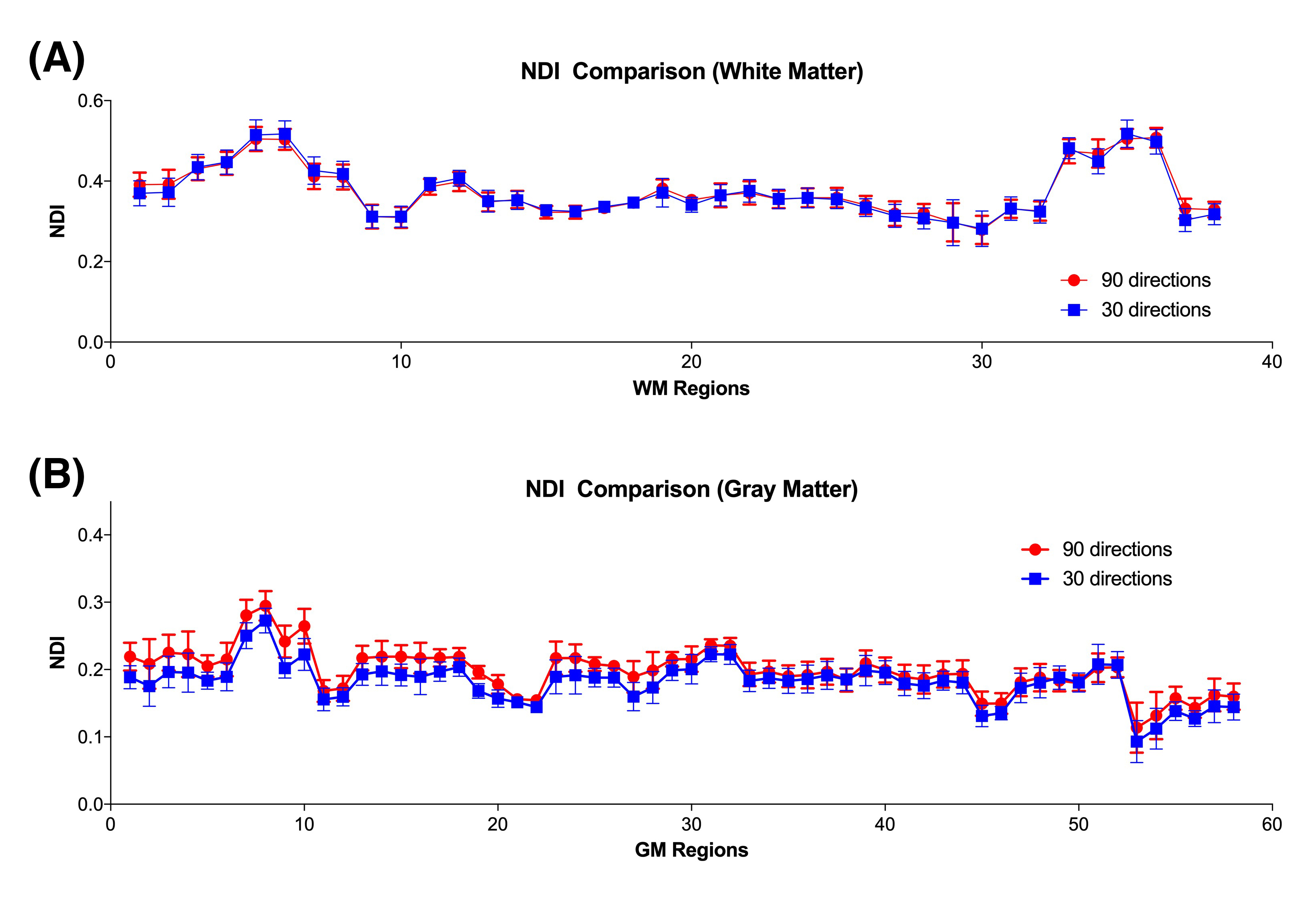

Supplement: Appendix Figure 4 — NDI comparison between protocol_30 and protocol_90. (A) The fitted NDI in 38 white matter (WM) regions. (B) The fitted NDI in 58 gray matter (GM) regions. [file Image_4.JPEG]

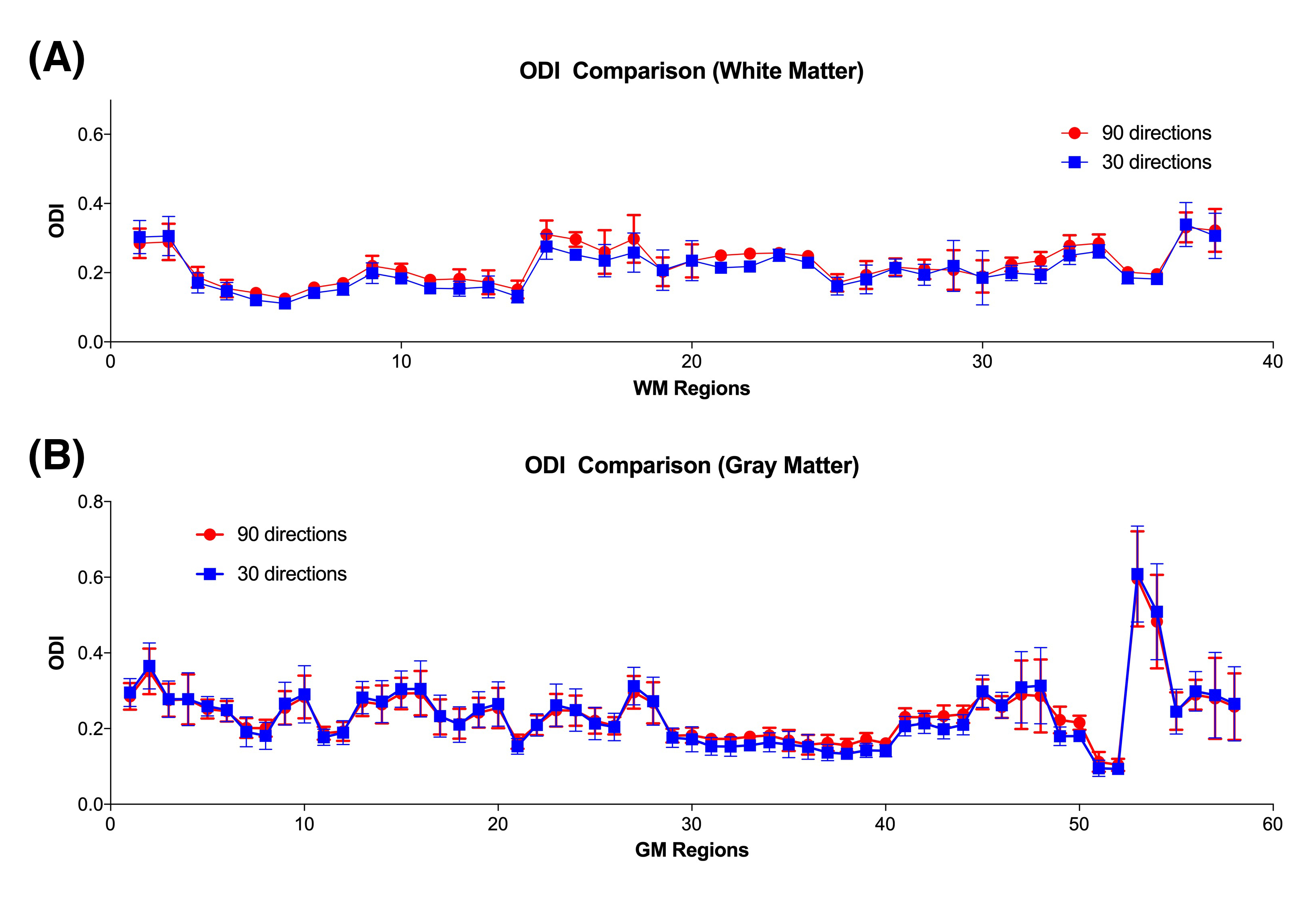

Supplement: Appendix Figure 5 — ODI comparison between protocol_30 and protocol_90. (A) The fitted ODI in 38 white matter (WM) regions. (B) The fitted ODI in 58 gray matter (GM) regions. [file Image_5.JPEG]
