# Supplementary material for: Brain Development From Newborn to Adolescence: Evaluation by Neurite Orientation Dispersion and Density Imaging
Source: Front Hum Neurosci. 2021 Mar 15;15:616132. doi: 10.3389/fnhum.2021.616132 (PMC8005551; doi:10.3389/fnhum.2021.616132)
Supplement: Appendix Table 1 — Exponential fitting results of NDI. * The order of brain regions remains the same as JHU Atlases (http://cmrm.med.jhmi.edu/). [file Table_1.pdf]

Appendix Table 1 Exponential Fitting Results of NDI

* The order of brain regions remains the same as JHU Atlases

(http://cmrm.med.jhmi.edu/)

122 brain regions			Fitting Results			
			**R-Square**	**C**	**A**	**τ/year**
1	corpus callosum	left	0.931	0.516	0.363	1.010
2	corpus callosum	right	0.940	0.532	0.365	1.112
3	anterior limb of internal capsule	left	0.946	0.535	0.352	1.613
4	anterior limb of internal capsule	right	0.943	0.514	0.335	1.248
5	posterior limb of internal capsule	left	0.923	0.619	0.347	1.495
6	posterior limb of internal capsule	right	0.925	0.623	0.353	1.587
7	retrolenticular part of internal capsule	left	0.922	0.496	0.310	1.134
8	retrolenticular part of internal capsule	right	0.930	0.506	0.328	1.286
9	anterior corona radiata	left	0.951	0.478	0.372	1.533
10	anterior corona radiata	right	0.956	0.474	0.368	1.478
11	superior corona radiata	left	0.952	0.551	0.417	1.304
12	superior corona radiata	right	0.953	0.544	0.413	1.106
13	posterior corona radiata	left	0.916	0.485	0.336	1.258
14	posterior corona radiata	right	0.916	0.472	0.330	1.175
15	cingulum cingular part	left	0.931	0.419	0.290	1.274
16	cingulum cingular part	right	0.935	0.402	0.275	0.984
17	cingulum hippocampal part	left	0.903	0.397	0.249	0.850
18	cingulum hippocampal part	right	0.914	0.395	0.248	0.763
19	fornix	left	0.483	0.317	0.173	1.936
20	fornix	right	0.762	0.400	0.254	2.306
21	stria terminalis	left	0.914	0.454	0.275	1.195
22	stria terminalis	right	0.913	0.415	0.240	1.053
23	tapetum	left	0.779	0.379	0.265	2.321
24	tapetum	right	0.773	0.323	0.229	0.816

25	superior longitudinal fasciculus	left	0.956	0.522	0.429	1.035
26	superior longitudinal fasciculus	right	0.953	0.533	0.442	1.106
27	external capsule	left	0.945	0.410	0.268	1.145
28	external capsule	right	0.948	0.434	0.289	1.292
29	posterior thalamic radiation	left	0.913	0.468	0.340	1.159
30	posterior thalamic radiation	right	0.913	0.457	0.325	1.176
31	sagittal stratum	left	0.933	0.432	0.307	1.396
32	sagittal stratum	right	0.936	0.432	0.309	1.280
33	thalamus	left	0.839	0.216	0.121	2.505
34	thalamus	right	0.839	0.196	0.110	1.405
35	putamen	left	0.929	0.194	0.117	1.791
36	putamen	right	0.928	0.189	0.112	1.677
37	globus pallidus	left	0.908	0.235	0.160	2.086
38	globus pallidus	right	0.914	0.239	0.161	2.196
39	caudate nucleus	left	0.788	0.154	0.110	2.832
40	caudate nucleus	right	0.773	0.140	0.095	1.636
41	cerebral peduncle	left	0.907	0.635	0.374	0.914
42	cerebral peduncle	right	0.902	0.644	0.370	1.454
43	superior fronto-occipital fasciculus	left	0.923	0.452	0.337	1.568
44	superior fronto-occipital fasciculus	right	0.896	0.397	0.272	0.991
45	inferior fronto-occipital fasciculus	left	0.955	0.420	0.275	1.521
46	inferior fronto-occipital fasciculus	right	0.955	0.421	0.277	1.425
47	corticospinal tract	left	0.876	0.634	0.375	0.946
48	corticospinal tract	right	0.877	0.626	0.376	0.982
49	superior cerebellar peduncle	left	0.878	0.521	0.234	1.036
50	superior cerebellar peduncle	right	0.884	0.514	0.250	0.808
51	middle cerebellar peduncle	left	0.792	0.617	0.395	0.382
52	middle cerebellar peduncle	right	0.807	0.606	0.393	0.423
53	inferior cerebellar peduncle	left	0.660	0.554	0.220	0.340
54	inferior cerebellar peduncle	right	0.628	0.524	0.196	0.260
55	pontine crossing tract	left	0.901	0.604	0.365	0.670
56	pontine crossing tract	right	0.906	0.579	0.362	0.600
57	uncinate fasciculus	left	0.888	0.359	0.214	0.719
58	uncinate fasciculus	right	0.912	0.358	0.211	0.794
59	midbrain	left	0.903	0.491	0.237	1.373
60	midbrain	right	0.899	0.476	0.224	1.271
61	pons	left	0.028	0.463	0.224	1.273
62	pons	right	0.759	0.491	0.219	1.272

63	medial lemniscus	left	0.806	0.530	0.213	0.629
64	medial lemniscus	right	0.816	0.525	0.203	0.922
65	medulla oblongata	left	0.499	0.462	0.157	0.307
66	medulla oblongata	right	0.314	0.447	0.143	0.205
67	superior frontal gyrus	left	0.847	0.149	0.111	1.807
68	superior frontal gyrus	right	0.811	0.135	0.098	1.553
69	middle frontal gyrus	left	0.859	0.152	0.115	1.418
70	middle frontal gyrus	right	0.881	0.158	0.116	1.666
71	inferior frontal gyrus	left	0.890	0.165	0.113	2.105
72	inferior frontal gyrus	right	0.920	0.176	0.129	2.500
73	medial fronto-orbital gyrus	left	0.851	0.166	0.118	1.002
74	medial fronto-orbital gyrus	right	0.823	0.170	0.120	1.033
75	lateral fronto-orbital gyrus	left	0.843	0.149	0.104	1.188
76	lateral fronto-orbital gyrus	right	0.870	0.153	0.107	1.218
77	gyrus rectus	left	0.890	0.146	0.097	1.828
78	gyrus rectus	right	0.865	0.145	0.096	1.491
79	precentral gyrus	left	0.864	0.183	0.128	2.215
80	precentral gyrus	right	0.889	0.188	0.129	2.351
81	postcentral gyrus	left	0.843	0.163	0.105	1.632
82	postcentral gyrus	right	0.800	0.152	0.094	1.133
83	superior parietal gyrus	left	0.883	0.204	0.142	2.206
84	superior parietal gyrus	right	0.892	0.201	0.140	2.046
85	precuneus	left	0.871	0.155	0.104	2.225
86	precuneus	right	0.817	0.134	0.086	1.588
87	cingular gyrus	left	0.922	0.150	0.093	2.114
88	cingular gyrus	right	0.865	0.124	0.075	1.076
89	supramarginal gyrus	left	0.866	0.160	0.107	1.680
90	supramarginal gyrus	right	0.874	0.166	0.110	1.897
91	angular gyrus	left	0.907	0.177	0.125	1.629
92	angular gyrus	right	0.907	0.165	0.115	1.126
93	superior temporal gyrus	left	0.901	0.148	0.109	2.154
94	superior temporal gyrus	right	0.912	0.151	0.111	2.041
95	middle temporal gyrus	left	0.951	0.161	0.116	1.157
96	middle temporal gyrus	right	0.948	0.168	0.121	1.467
97	inferior temporal gyrus	left	0.907	0.157	0.106	1.229
98	inferior temporal gyrus	right	0.912	0.156	0.110	1.037
99	fusiform gyrus	left	0.906	0.161	0.103	2.010
100	fusiform gyrus	right	0.874	0.160	0.102	1.886
101	parahippocampal gyrus	left	0.698	0.203	0.150	10.970
102	parahippocampal gyrus	right	0.714	0.147	0.095	4.728
103	entorhinal cortex	left	0.527	0.113	0.077	2.545

104	entorhinal cortex	right	0.605	0.128	0.086	2.199
105	superior occipital gyrus	left	0.913	0.209	0.149	2.237
106	superior occipital gyrus	right	0.903	0.209	0.150	1.719
107	middle occipital gyrus	left	0.929	0.186	0.129	1.350
108	middle occipital gyrus	right	0.938	0.187	0.130	1.314
109	inferior occipital gyrus	left	0.924	0.172	0.118	1.100
110	inferior occipital gyrus	right	0.938	0.180	0.122	1.327
111	cuneus	left	0.882	0.170	0.110	1.532
112	cuneus	right	0.895	0.174	0.112	1.699
113	lingual gyrus	left	0.895	0.178	0.113	2.131
114	lingual gyrus	right	0.897	0.178	0.116	1.996
115	amygdala	left	0.816	0.139	0.066	2.906
116	amygdala	right	0.791	0.140	0.063	3.388
117	hippocampus	left	0.850	0.129	0.076	1.494
118	hippocampus	right	0.832	0.129	0.076	1.511
119	cerebellar hemisphere	left	0.851	0.457	0.238	0.273
120	cerebellar hemisphere	right	0.827	0.462	0.232	0.288
121	insular cortex	left	0.801	0.113	0.067	2.261
122	insular cortex	right	0.852	0.126	0.074	2.894
